# Evaluating the Diagnostic Potential of the FIB-4 Index for Cystic Fibrosis-Associated Liver Disease in Adults: A Comparison with Transient Elastography

**DOI:** 10.3390/jcm14155404

**Published:** 2025-07-31

**Authors:** Stephen Armstrong, Kingston Rajiah, Aaron Courtenay, Nermeen Ali, Ahmed Abuelhana

**Affiliations:** 1 Northern Ireland Regional Cystic Fibrosis Centre, Belfast Health and Social Care Trust, Belfast BT12 6BA, UK; stephen_armstrong1990@hotmail.com; 2 School of Pharmacy and Pharmaceutical Sciences, Ulster University, Coleraine BT52 1SA, UK; a.courtenay@ulster.ac.uk (A.C.); n.ali@ulster.ac.uk (N.A.); a.abuelhana@ulster.ac.uk (A.A.)

**Keywords:** cystic fibrosis, cystic fibrosis-associated liver disease (CFLD), Fibrosis-4 index (FIB-4), transient elastography (TE), liver fibrosis, non-invasive diagnostics, liver stiffness measurement, CFTR modulators

## Abstract

**Background/Objectives**: Cystic fibrosis-associated liver disease (CFLD) is a significant complication in individuals with cystic fibrosis (CF), contributing to morbidity and mortality, with no universally accepted, reliable, non-invasive diagnostic tool for early detection. Current diagnostic methods, including liver biopsy and imaging, remain resource-intensive and invasive. Non-invasive biomarkers like the Fibrosis-4 (FIB-4) index have shown promise in diagnosing liver fibrosis in various chronic liver diseases. This study explores the potential of the FIB-4 index to predict CFLD in an adult CF population and assesses its correlation with transient elastography (TE) as a potential diagnostic tool. The aim of this study is to evaluate the diagnostic performance of the FIB-4 index for CFLD in adults with CF and investigate its relationship with TE-based liver stiffness measurements (LSM). **Methods**: The study was conducted in a regional cystic fibrosis unit, including 261 adult CF patients. FIB-4 scores were calculated using an online tool (mdcalc.com) based on patient age, aspartate aminotransferase (AST), alanine aminotransferase (ALT), and platelet count. In parallel, 29 patients underwent liver stiffness measurement using TE (Fibroscan^®^). Statistical analyses included non-parametric tests for group comparisons and Pearson’s correlation to assess the relationship between FIB-4 scores and TE results. **Results**: The mean FIB-4 score in patients diagnosed with CFLD was higher (0.99 ± 0.83) compared to those without CFLD (0.64 ± 0.38), although the difference was not statistically significant (*p* > 0.05). TE results for CFLD patients (5.9 kPa) also did not show a significant difference compared to non-CFLD patients (4.2 ± 1.6 kPa, *p* > 0.05). However, a positive correlation (r = 0.401, *p* = 0.031) was found between FIB-4 scores and TE-based LSM, suggesting a potential complementary diagnostic role. **Conclusions**: The FIB-4 index, while not sufficient as a standalone diagnostic tool for CFLD in adults with CF, demonstrates potential when used in conjunction with other diagnostic methods like TE. This study introduces a novel approach for integrating non-invasive diagnostic markers in CF care, offering a pathway for future clinical practice. The combination of FIB-4 and TE could serve as an accessible, cost-effective alternative to invasive diagnostic techniques, improving early diagnosis and management of CFLD in the CF population. Additionally, future research should explore the integration of these tools with emerging biomarkers and clinical features to refine diagnostic algorithms for CFLD, potentially reducing reliance on liver biopsies and improving patient outcomes.

## 1. Introduction

Cystic fibrosis (CF) is the most common autosomal recessive disease among the Caucasian population, with recent global estimates indicating over 160,000 diagnosed individuals worldwide, according to international registry data [1]. The disease is particularly common in Europe, North America, and Australia, while lower prevalence is reported in Asia and sub-Saharan Africa due to underdiagnosis or genetic variability. Advances in neonatal screening and CF care have significantly improved life expectancy, with many individuals now living into adulthood and beyond, thus highlighting the importance of managing long-term complications, including those affecting the liver [1].

CF is a progressive, multisystem disorder that results from mutations in the cystic fibrosis transmembrane conductance regulator (CFTR) gene [2]. The CFTR protein functions primarily as a chloride channel in epithelial cells, regulating ion and water transport across cell membranes [3]. Mutations in this gene lead to the production of a dysfunctional or absent CFTR protein, impairing chloride transport and resulting in the accumulation of dehydrated, viscous mucus in various organs [3]. This pathological mucus affects primarily the respiratory and gastrointestinal systems, but it also contributes to complications in the liver, pancreas, and reproductive tract.

More than 1900 CFTR mutations have been identified, and the severity of CF varies depending on the specific mutation(s) inherited [4]. CFTR mutations are categorised into six classes based on their functional impact, ranging from complete absence of CFTR protein (Class I) to defective regulation or decreased stability (Classes IV–VI). Individuals with two severe mutations, typically in Classes I–III, often exhibit classic CF symptoms, including pancreatic insufficiency and a higher risk for CFLD [4]. Common features include chronic pulmonary infections, bronchiectasis, progressive loss of lung function, pancreatic insufficiency, malnutrition, CF-related diabetes, infertility, and cystic fibrosis-associated liver disease (CFLD) [5].

While pulmonary complications are the leading cause of morbidity and mortality in CF, extrapulmonary manifestations like CFLD are gaining increasing recognition due to the improvements in respiratory management that have extended patient survival. CFLD is now the third most common cause of death among individuals with CF [6]. It affects up to 40% of people with CF, with liver abnormalities typically emerging in childhood or adolescence [7]. The natural history of CFLD is insidious, and many patients remain asymptomatic until substantial hepatic damage has occurred.

The pathophysiology of CFLD is complex and not yet fully elucidated. It is hypothesised that CFTR dysfunction leads to alterations in bile composition and impaired bile excretion, resulting in biliary stasis, ductal injury, and chronic hepatic inflammation [8]. Over time, this process contributes to fibrosis, nodular regeneration, and eventually cirrhosis. However, not all individuals with CF develop liver disease, suggesting that genetic modifiers, environmental factors, and comorbidities such as pancreatic insufficiency or nutritional deficiencies may also influence disease progression [9].

In some patients, progressive liver fibrosis may lead to the development of portal hypertension, which manifests clinically as splenomegaly, variceal bleeding, or hypersplenism. The occurrence of such complications significantly impacts the quality of life and complicates the overall management of CF. Furthermore, the increasing use of highly effective CFTR modulators, such as ivacaftor and the triple combination elexacaftor/tezacaftor/ivacaftor, is changing the landscape of CF care. These therapies have shown promise in reducing pulmonary exacerbations and improving nutritional status, but their long-term impact on hepatic manifestations remains under investigation. Some case reports suggest potential hepatotoxicity, while others hypothesise a possible stabilising effect on bile composition [7,8].

Diagnosing CFLD is challenging because early-stage liver disease is typically subclinical. By the time physical signs such as hepatomegaly, splenomegaly, or complications like portal hypertension appear, irreversible liver damage may have already occurred [8]. Furthermore, there is no single, universally accepted test for diagnosing CFLD [10]. European guidelines recommend routine screening using liver biochemistry, clinical examination, and abdominal ultrasonography [11]. These tools, while useful, lack sensitivity and specificity, particularly for early disease detection. Liver biopsy, although historically considered the gold standard for diagnosing liver fibrosis and cirrhosis, has several limitations in the context of CFLD, including its invasive nature, sampling error due to the focal nature of liver involvement, and the risk of complications [12].

CFLD is typically diagnosed based on a combination of clinical, biochemical, radiological, and histological findings. At least two of the following four criteria must be met for diagnosis: clinical signs such as hepatosplenomegaly, biochemical abnormalities like persistently elevated liver enzymes (e.g., Aspartate transaminase (AST), Alanine transaminase (ALT), Gamma-glutamyl transferase (GGT), radiologic evidence from ultrasound (e.g., signs of portal hypertension or biliary tract abnormalities), or histological confirmation of fibrosis or cirrhosis [13]. Due to the limited reliability of these modalities when used in isolation, there is a growing interest in identifying alternative non-invasive diagnostic methods that can facilitate earlier detection and monitoring.

One such technique is transient elastography (TE), a non-invasive imaging modality that quantifies liver stiffness using shear wave velocity measurements [14]. TE has been extensively validated in various chronic liver diseases, including hepatitis C, non-alcoholic fatty liver disease (NAFLD), and primary sclerosing cholangitis. Its advantages include speed, repeatability, patient comfort, and avoidance of biopsy-related risks [15]. In CF, TE shows promise for detecting hepatic fibrosis and monitoring disease progression, yet its availability may be limited by the need for specialist equipment (e.g., Fibroscan^®^) and operator training. Moreover, the accuracy of TE in CF may be reduced due to the patchy, heterogeneous distribution of fibrosis typical in CFLD. In clinical practice, the interpretation of TE results should consider potential confounding factors such as inflammation, cholestasis, and hepatic congestion, which can falsely elevate liver stiffness measurements.

In addition to imaging, several biochemical scoring systems have been developed to assess liver fibrosis in non-CF populations [16]. One of the most widely studied is the Fibrosis-4 (FIB-4) index, which combines age, AST, ALT, and platelet count into a simple formula [17]. Originally developed to assess hepatic fibrosis in patients with viral hepatitis, the FIB-4 index has demonstrated utility in identifying advanced fibrosis in a variety of liver diseases, including NAFLD and alcoholic liver disease [16]. Because it relies on commonly available laboratory parameters, FIB-4 has become an attractive screening tool in both primary and secondary care settings. Although the FIB-4 index is age-dependent and was initially validated in older populations with chronic liver diseases, its simplicity and accessibility make it a potentially useful comparative tool within adult CF cohorts, particularly when used alongside other diagnostic modalities.

In other chronic liver diseases, the FIB-4 index has been incorporated into clinical algorithms to reduce unnecessary biopsies and facilitate referral to hepatology specialists. For example, a low FIB-4 score may rule out significant fibrosis and support continued monitoring in primary care, whereas an elevated FIB-4 score might prompt further evaluation with TE or magnetic resonance elastography. However, there is limited literature evaluating the applicability of the FIB-4 index in the CF population. Given the unique pathophysiological features of CFLD and the potential for overlapping biochemical abnormalities related to other CF complications, such as chronic infection or nutritional deficiencies, the accuracy and clinical relevance of FIB-4 in this context remain uncertain. Moreover, the potential effect of CFTR modulators on transaminase levels may further complicate interpretation.

Despite these challenges, the simplicity and accessibility of the FIB-4 index make it a promising candidate for inclusion in non-invasive CFLD screening protocols, particularly in resource-limited settings or where TE is unavailable. The potential synergy between FIB-4 and TE lies in their complementary mechanisms—FIB-4 reflects systemic changes in liver function, while TE offers a direct measure of parenchymal stiffness. Used together, they may improve diagnostic accuracy and inform timely intervention strategies.

To date, very few studies have systematically compared FIB-4 with TE in adult CF populations. Determining whether a correlation exists between FIB-4 scores and liver stiffness measurements (LSMs) from TE could validate the FIB-4 index as a surrogate marker for liver involvement in CF. If proven reliable, this could allow earlier identification of at-risk individuals, facilitate closer monitoring, and potentially reduce the need for invasive procedures such as liver biopsy. There is also scope for future integration of artificial intelligence and machine learning algorithms to optimise the interpretation of FIB-4 and TE data, particularly in longitudinal monitoring.

This study was designed as a service improvement initiative aimed at assessing the clinical utility of the FIB-4 index as a non-invasive marker for CFLD in an adult CF population in Northern Ireland. A secondary objective was to determine whether FIB-4 scores correlate with TE-derived LSM values, thereby evaluating the potential complementary role of these tools in CF liver disease management. By exploring these parameters, this study seeks to contribute to the ongoing search for accurate, accessible, and non-invasive methods for diagnosing and monitoring CFLD, ultimately supporting improved patient care in this vulnerable population.

## 2. Materials and Methods

### 2.1. Study Setting

The study was conducted at the regional cystic fibrosis unit in Belfast City Hospital, which is part of the Belfast Health and Social Care Trust (BHSCT). All blood tests, data analysis, and LSMs were performed within this facility.

### 2.2. Study Design

This was a retrospective observational study conducted over 18 weeks from May to September 2023. Data collection was completed within the first seven weeks, allowing time for TE assessments to be conducted and analysed. Inclusion criteria encompassed persons with cystic fibrosis (PWCFs) aged 18–65 years who had undergone a full blood count and liver function test within the preceding year. Exclusion criteria included patients older than 65 years and those who had undergone liver transplantation. Patients over 65 years of age were excluded to maintain a representative adult CF population and to minimise confounding from age-related liver changes unrelated to CF. Additionally, as age is a significant component of the FIB-4 score calculation, inclusion of older individuals could disproportionately influence fibrosis score interpretation within the study cohort.

A total of 287 PWCFs were screened, and 261 were included in the study based on the inclusion criteria for FIB-4 score calculations, while 29 TE assessments were performed. The sample size was based on the average number of patients attending the CF unit of Belfast City Hospital.

### 2.3. Data Collection

Diagnosis of CFLD in the study population was based on documented clinical diagnosis recorded in the electronic care records by the treating clinicians. This diagnosis was made according to standard clinical practice within the CF unit, typically incorporating a combination of clinical findings, biochemical markers, and imaging results in accordance with established guidelines. As this was a retrospective study, no additional diagnostic assessment was performed by the study investigators. Data collection was conducted in two stages. Initially, electronic care records of persons with cystic fibrosis (PWCFs) were reviewed to calculate FIB-4 scores. In the second stage, TE assessments were performed, and the data were anonymised before being passed on to the investigator for analysis. A centralised data collection spreadsheet was used to securely compile the information.

Electronic records for 261 PWCFs were reviewed by one of the researchers to extract relevant laboratory results, including age, AST, ALT, and platelet count. These values were used to calculate FIB-4 scores using the online tool on mdcalc.com. The resulting scores were categorised into three fibrosis stages: mild (Ishak fibrosis score 0–1), moderate (Ishak fibrosis score 2–3), and advanced (Ishak fibrosis score 4–6). Significant fibrosis was defined as an Ishak fibrosis score of 4 or greater, and cirrhosis was defined as a score of 5 or 6. In parallel, a nurse conducted TE assessments on eligible patients using a Fibroscan^®^ device. In our regional CF unit, transient elastography assessments are routinely performed by a trained CF nurse practitioner. The nurse conducting TE in this study had undergone certified training provided by the FibroScan^®^ device manufacturer (Echosens, Paris, France), including hands-on competency assessment, in accordance with local clinical protocols. This practice aligns with standard operating procedures within the unit for non-invasive liver stiffness assessment. The TE values were compared against a cut-off value of ≥5.95 kPa [18]. TE results included in this study were obtained retrospectively from clinical records, where TE was performed as part of routine clinical practice at the regional CF unit. TE assessments were conducted using the FibroScan^®^ device by trained personnel following standardised protocols. According to unit practice, patients typically underwent TE in the fasting state, with the M-probe used for standard adult assessments.

### 2.4. Statistical Analysis

All data were anonymised before analysis. The normality of data distribution was assessed using the Kolmogorov–Smirnov test for sample sizes greater than 10 and the Shapiro–Wilk test for sample sizes of 10 or fewer. Data that did not meet the assumptions for normality (*p* < 0.05) were analysed using non-parametric methods, such as the Mann–Whitney U test for comparing two independent groups and the Kruskal–Wallis test for comparisons across more than two groups. For normally distributed data, parametric tests were applied, including independent t-tests for two-group comparisons and one-way ANOVA for comparing more than two groups. Pearson’s correlation coefficient was used to assess the strength and direction of linear relationships between continuous variables, such as FIB-4 scores and LSMs (TE results). Descriptive statistics, including means and standard deviations (SDs) for continuous variables and proportions for categorical variables, were reported where appropriate. All analyses were performed using SPSS software (version 26), and statistical significance was set at *p* < 0.05.

### 2.5. Data Security

Patient identifiers were excluded from the dataset to ensure anonymity. All data were stored securely on a password-protected computer accessible only to authorised investigators.

## 3. Results

The study included 261 PWCFs who had their FIB-4 scores calculated. Of these, 29 individuals underwent TE assessments. The baseline demographic and clinical characteristics are presented in Table 1.

### 3.1. FIB-4 Scores

FIB-4 scores were categorised into fibrosis stages, with the distribution shown in Table 2. The Mann–Whitney U test was used because the FIB-4 score distribution was found to be non-normal, as indicated by the Kolmogorov–Smirnov test. However, the difference in scores was not statistically significant. The majority of patients in both groups were classified as having mild fibrosis based on FIB-4 scores, with 200 out of 234 (85.5%) non-CFLD patients and 14 out of 27 (51.9%) CFLD patients falling into the mild fibrosis category. In contrast, moderate fibrosis was observed in 30 non-CFLD patients (12.8%) and 10 CFLD patients (37.0%). Advanced fibrosis was identified in only a small proportion of patients: four non-CFLD patients (1.7%) and three CFLD patients (11.1%). These findings reflect a predominance of lower fibrosis scores in the overall CF population, with a trend toward higher fibrosis stages in the CFLD group.

### 3.2. Transient Elastography Results

Characteristics of the TE-assessed population in this study are shown in Table 3. A total of 29 patients underwent TE assessments, with only 1 diagnosed CFLD case. The mean LSM was 5.9 kPa for the CFLD patient and 4.2 ± 1.6 kPa for patients without CFLD. The independent t-test was used as the LSM data appeared to be normally distributed, but the difference in mean LSM between these groups was not statistically significant. The results highlight key demographic and clinical characteristics of these patients, revealing that the single CFLD patient undergoing TE exhibited higher liver stiffness values compared to the mean values of non-CFLD patients. The results also show a higher proportion of pancreatic insufficiency and CFTR modulator use among CFLD patients, reflecting the clinical complexity of managing such cases. These findings suggest that while TE can capture variations in liver stiffness, its utility in isolating CFLD-specific patterns requires larger and more balanced samples to ensure reliability in clinical interpretations.

**Table 3:** Characteristics of TE-assessed population.

Characteristic	TE Population (n = 29)	CFLD (n = 1)	No CFLD (n = 28)
Mean Age (years ± SD)	36.1 ± 12.3	42	36.0 ± 12.2
Gender (% male)	51.7%	100%	50.0%
Homozygous F508del (%)	20.7%	0%	21.4%
Prescribed CFTR Modulator (%)	72.4%	100%	71.4%
Pancreatic Insufficient (%)	51.7%	100%	50.0%

The correlation between FIB-4 scores and LSMs is shown in Table 4. Pearson’s correlation coefficient analysis demonstrated a slight positive correlation between FIB-4 scores and LSMs (r = 0.401, *p* = 0.031).

**Table 4:** Correlation between FIB-4 scores and LSM results.

Variable 1	Variable 2	Correlation Coefficient (r)	*p*-Value
FIB-4 Score	LSM	0.401	0.031 *

* *p* < 0.05.

## 4. Discussion

CFLD represents a significant source of morbidity and mortality among individuals with CF. Despite improvements in respiratory care and the introduction of CFTR modulators, hepatic complications remain a considerable clinical burden. The literature indicates that CFLD often manifests early in life, yet its asymptomatic nature in the initial stages makes diagnosis challenging and often delayed [19]. The pathogenesis of CFLD is complex and multifactorial, involving altered bile composition, impaired bile flow due to CFTR dysfunction, chronic inflammation, and susceptibility conferred by specific genetic mutations [20,21]. These factors underscore the need for more nuanced, non-invasive diagnostic approaches that can detect liver damage before it progresses to irreversible fibrosis or cirrhosis.

Emerging diagnostic frameworks suggest integrating standardised biochemical indices like the Fibrosis-4 (FIB-4) index and imaging techniques such as transient elastography (TE) to improve diagnostic accuracy in CFLD. Recent research advocates complementing these methods with disease-specific markers, including serum bile acids, hyaluronic acid, and proinflammatory cytokines, to reflect the systemic and hepatic inflammatory processes unique to CF [22,23]. TE has earned attention due to its non-invasive nature and reliability in assessing liver fibrosis in other chronic liver conditions, including hepatitis B and C, and non-alcoholic fatty liver disease. However, its clinical utility in CF remains under scrutiny due to the focal and heterogeneous nature of fibrosis associated with CFLD [24]. Therefore, a multidimensional diagnostic strategy is increasingly viewed as essential for managing liver disease in CF.

The demographic and clinical features of CF patients play a critical role in disease progression and the risk of developing CFLD. Previous studies have suggested that males may have a slightly higher predisposition to liver complications, possibly due to sex-related hormonal or immunologic factors that modulate bile acid metabolism and hepatic inflammation [25]. CFTR genotypes also influence disease severity, with the F508del mutation associated with a higher likelihood of liver involvement [26]. Pancreatic insufficiency, frequently observed in patients with CFLD, exacerbates malabsorption and nutritional deficiencies, further impairing hepatic function [27]. These patient-specific characteristics highlight the importance of adopting an individualised approach to CFLD diagnosis and monitoring, tailored to the genetic and clinical profile of each patient.

Patients diagnosed with CFLD typically present with more advanced fibrosis compared to non-CFLD individuals, which aligns with the broader understanding that CFLD progresses silently until substantial hepatic injury has occurred. Clinically, this highlights the importance of early screening using tools sensitive enough to detect preclinical changes. Traditional fibrosis scores such as the FIB-4 index have proven valuable in other liver diseases but are potentially limited in CF due to unique hepatic pathologies, including irregular bile flow and localised inflammation [7]. Nonetheless, elevated FIB-4 scores in CFLD patients are consistent with a higher degree of hepatic insult. In contrast, non-CFLD patients typically display lower fibrosis stages, reflecting the variability in hepatic involvement across the CF population. These patterns support the utility of integrating routine fibrosis scoring systems into CF care but also suggest that such tools should be used alongside CF-specific clinical indicators.

Moreover, variations in hepatic involvement among CF patients further highlight the heterogeneity of disease progression. Some individuals may present with biochemical abnormalities but no radiological findings, while others may show radiological signs without abnormal blood tests. This reinforces the inadequacy of relying on a single diagnostic tool and the necessity of composite approaches. Furthermore, clinicians must remain aware of transient enzyme elevations that can occur during pulmonary exacerbations or due to medications, which may confound FIB-4 interpretation. These clinical nuances necessitate a careful, context-specific analysis of all available diagnostic data.

The clinical utility of TE in assessing liver stiffness offers significant advantages, especially in patients who are poor candidates for liver biopsy. In this study, patients with suspected CFLD who underwent TE exhibited higher liver stiffness values than those without CFLD, in accordance with the existing literature. This trend reflects the multifactorial liver injury in CF, where bile duct obstruction, inflammation, and fibrosis coalesce to elevate stiffness readings. Moreover, this patient subgroup also demonstrated higher incidences of pancreatic insufficiency and CFTR modulator use, suggesting a more severe disease phenotype. Although TE offers valuable insights, its restrictions, especially in detecting patchy fibrosis, mean that it is best used in conjunction with biochemical markers and clinical assessment to ensure diagnostic accuracy.

TE may also be influenced by other factors such as hepatic congestion, steatosis, and inflammation, which can result in overestimation or underestimation of liver stiffness [28]. These confounding factors, coupled with the focal nature of CFLD lesions, reduce the standalone reliability of TE. In some cases, TE may fail to detect early perisinusoidal fibrosis, which can still have significant clinical implications. Hence, while TE is an important non-invasive tool, its results should be interpreted within the broader clinical context.

The combined use of FIB-4 and TE may overcome some of the individual limitations of each method. While FIB-4 is easily calculated and accessible, its utility in CF is constrained by the underlying disease’s influence on liver enzymes and platelet counts. TE, although more direct in its assessment of fibrosis, is limited by the cost, availability, and requirement for operator training. The literature supports that these tools, when used together, provide a more robust picture of liver health [29]. A synergistic diagnostic approach—where FIB-4 may serve as a screening tool and TE as a confirmatory measure—could facilitate earlier intervention and better risk stratification. Some studies have also proposed adding novel serum biomarkers or imaging modalities to this algorithm to enhance sensitivity and specificity in CFLD detection [30,31].

Given the chronic, progressive, and often silent nature of CFLD, regular monitoring using non-invasive methods is essential. Patients showing elevated FIB-4 scores or abnormal TE results should be prioritised for more frequent follow-up or specialist hepatology referral. Integrating these tools into standard CF management protocols may offer a practical alternative to invasive liver biopsy, reducing patient burden and enabling proactive care.

### 4.1. Clinical Implications

The potential clinical implications of this study are considerable. By supporting the complementary use of FIB-4 and TE, the study promotes a shift toward a more holistic, non-invasive screening paradigm in CF hepatology. This could lead to earlier detection of liver disease, optimisation of treatment plans, and improved patient outcomes. It also encourages further investigation into how non-invasive methods can be customised for use in CF populations, considering their unique pathophysiological traits. The application of such tools could be extended to community or outpatient settings, reducing the need for tertiary centre referral and expanding access to liver disease screening. Furthermore, this study aligns with the broader movement toward personalised medicine in CF care. As genetic, environmental, and treatment-related variables contribute to disease heterogeneity, diagnostic tools must also be adapted to reflect these complexities. Non-invasive tools such as FIB-4 and TE could be incorporated into patient-specific monitoring plans, offering clinicians a tailored approach to managing hepatic complications. This is particularly relevant in the era of CFTR modulator therapies, where long-term effects on extrapulmonary systems, including the liver, remain an area of active investigation.

### 4.2. Limitations

Several limitations must be acknowledged. The sample size for TE assessments was relatively small, particularly for CFLD patients, which limits statistical power and generalisability. Additionally, the retrospective nature of the FIB-4 component introduces potential biases related to data completeness and variability in the timing of blood sampling. The study was also confined to a single regional CF centre, which may not reflect wider CF population diversity. The unavailability of comprehensive longitudinal data restricted the analysis of disease progression. Furthermore, emerging confounding variables such as the introduction of CFTR modulators and their potential hepatic effects were not fully explored in this cohort. Another important limitation is the lack of liver biopsy data to validate the non-invasive markers. Histological confirmation remains the definitive method for assessing liver fibrosis; however, ethical and practical considerations limit its use. The absence of histological data in this study restricts the ability to draw definitive conclusions about the accuracy of FIB-4 and TE in detecting true fibrosis stages. The phenotypic characterisation based on cirrhotic versus non-cirrhotic status, as recommended by the North American and European Societies for Paediatric Gastroenterology, Hepatology and Nutrition, was not systematically recorded in the patient records and therefore was not applied in this study. Patients were classified as having CFLD based on documented clinical diagnosis at the time of data collection. Specific radiological findings on abdominal ultrasound (e.g., steatosis, evidence of portal hypertension) were not consistently recorded in patient records and could not be subdivided further in this analysis. Future studies incorporating biopsy correlation would be instrumental in validating these non-invasive tools.

### 4.3. Future Directions

Despite these limitations, this study reinforces the potential value of combining non-invasive diagnostic tools in the management of CFLD. Neither FIB-4 nor TE alone proved definitive in identifying CFLD, yet their complementary use appears promising. Moving forward, a composite diagnostic algorithm incorporating TE, FIB-4, and CF-specific biomarkers such as bile acids and inflammatory cytokines may improve diagnostic accuracy. Additionally, shear wave elastography (SWE), another non-invasive ultrasound-based technique, has emerged as a promising tool for assessing liver stiffness in chronic liver diseases. Unlike TE, SWE allows for real-time imaging and the assessment of specific liver regions, potentially improving detection of the patchy fibrosis often seen in CFLD. Future studies should explore the applicability and diagnostic performance of SWE in the CF population.

Furthermore, recent work by Dana et al. [32] has highlighted the changing landscape of CFLD monitoring in the era of CFTR modulator therapies. These therapies may influence the progression of liver disease and alter traditional biomarkers, thereby impacting both biochemical and imaging-based assessments. Dana et al. advocate for integrating non-invasive diagnostic methods, including TE and fibrosis scores, with vigilant clinical monitoring to adapt to the dynamic disease course associated with CFTR modulators. This aligns with the approach explored in our study, highlighting the need for flexible diagnostic strategies in modern CF care. Future research should focus on multicentre prospective studies with larger and more diverse cohorts. Longitudinal designs would allow for tracking the evolution of liver disease and the predictive validity of non-invasive markers over time. It would also be beneficial to investigate how CFTR modulators influence hepatic parameters and whether their use modifies the diagnostic performance of FIB-4 or TE. Ultimately, the goal should be to develop a comprehensive, patient-centred, non-invasive diagnostic strategy that improves early detection, facilitates timely intervention, and reduces dependence on liver biopsy in the routine care of individuals with CF. By refining and validating such strategies, clinicians can improve outcomes for CF patients at risk of CFLD, helping to maintain liver health as an integral component of long-term disease management.

## 5. Conclusions

This study aimed to evaluate the diagnostic potential of the Fibrosis-4 (FIB-4) index and transient elastography (TE) for identifying cystic fibrosis-associated liver disease (CFLD) in adults with cystic fibrosis (CF). While the results demonstrated that neither FIB-4 nor TE is sufficient as a standalone diagnostic tool, their complementary use shows promise. FIB-4 offers a simple, non-invasive approach to estimating liver fibrosis, whereas TE provides direct measurement of liver stiffness, albeit with limitations in detecting focal or heterogeneous fibrosis typical of CFLD. The findings suggest that integrating FIB-4 and TE into a diagnostic framework alongside CF-specific clinical and biochemical markers could enhance early detection and management of CFLD. This combined approach may help identify at-risk patients, allowing for timely interventions and improved clinical outcomes. However, the small sample size for TE and the restricted representation of CFLD cases highlight the need for larger, multicentre studies to validate these results and establish a reliable, non-invasive diagnostic algorithm for CFLD in CF populations.

## Figures and Tables

**Table 1:** Demographic and clinical characteristics of study population.

Characteristic	Total (n = 261)	CFLD (n = 27)	No CFLD (n = 234)
Mean Age (years ± SD)	34.6 ± 10.7	32.2 ± 12.6	34.8 ± 10.4
Mean Weight (kg ± SD)	71.3 ± 15.1	-	-
Mean ppFEV_1_ (% ± SD)	76.1 ± 22.3	-	-
Homozygous F508del (%)	35.2%	55.6%	32.9%
Prescribed CFTR Modulator (%)	81.6%	88.9%	80.8%
Pancreatic Insufficient (%)	66.3%	81.5%	64.5%
Abnormal Ultrasound (%)	-	81.5%	23.5%

ppFEV_1_—percent predicted forced expiratory volume.

**Table 2:** Distribution of FIB-4 scores by fibrosis stage.

Fibrosis Stage	CFLD (n = 27)	No CFLD (n = 234)
Mild (0–1 Ishak)	14	200
Moderate (2–3 Ishak)	10	30
Advanced (4–6 Ishak)	3	4

## Data Availability

The data presented in the study are stored securely at the School of Pharmacy and Pharmaceutical Sciences, Ulster University. Investigators act as custodians for the data processed and generated by the study, and they are also responsible for access to any information included. The data are available upon request from the corresponding author. Due to privacy and institutional regulations, the data are not publicly accessible.
